# Restrictive Strabismus Following Frontotemporal-orbitozygomatic Craniotomy

**DOI:** 10.7759/cureus.1937

**Published:** 2017-12-11

**Authors:** Oluwatobi O Idowu, Evan Kalin-Hajdu, F Lawson Grumbine, Robert C Kersten, Michael McDermott, M Reza Vagefi

**Affiliations:** 1 Ophthalmology, University of California, San Francisco; 2 Ophthalmology, University of Maryland; 3 Department of Neurological Surgery, University of California, San Francisco

**Keywords:** sphenoid wing meningioma, restrictive strabismus, orbital reconstruction, forced duction testing, lateral rectus, diplopia, frontotemporal-orbitozygomatic craniotomy

## Abstract

The frontotempotal-orbitozygomatic craniotomy (FTOZ) is a standard approach for large sphenoid wing meningiomas (SWMs). Nevertheless, resection of these tumors is not without ophthalmologic risks. This series presents two patients with acute postoperative restrictive strabismus following tumor resection and orbital wall reconstruction. Forced duction testing and postoperative imaging revealed impingement of the lateral rectus muscle caused by an alloplastic implant and/or residual bone, prompting immediate orbitotomy and restoration of normal extraocular muscle function. This report highlights the intricacies of orbital reconstruction, as well as the need for intraoperative forced duction testing.

## Introduction

Sphenoid wing meningiomas (SWMs) are, most-often, benign neoplasms that represent approximately 15-20% of all intracranial meningiomas and comprise 2% of all orbital tumors [1-2]. Growth of an SWM can result in compressive optic neuropathy, other adjacent cranial neuropathies, as well as compromise of the cavernous sinus portion of the internal carotid artery. Historically, surgical resection has proven to be challenging; however, the standard use of the frontotemporal-orbitozygomatic (FTOZ) craniotomy has now greatly improved neurosurgical access for complete, or near complete, tumor removal. At our institution, the surgical technique involves a multidisciplinary approach with neurosurgery and oculoplastic surgery when there is significant bony involvement of the roof and lateral wall of the orbit [3]. After tumor resection, the orbital roof and lateral wall are reconstructed with an alloplastic implant.

This report describes two patients who developed binocular horizontal diplopia secondary to lateral rectus muscle restriction following FTOZ craniotomy and orbital wall reconstruction. It highlights the importance of intraoperative forced duction testing to avoid this complication and the need for urgent surgical revision should the complication arise. The case review was conducted in full compliance with the provisions of the Health Insurance Portability and Accountability Act (HIPAA) of 1996 and consent was obtained for clinical photographs.

## Case presentation

Case 1

A 57-year-old woman with World Health Organization (WHO) grade I SWM presented due to a six-month history of progressive deterioration in visual acuity (VA) of the right eye (OD). An examination revealed best-corrected visual acuity (BCVA) of 20/50 OD, 4 mm of right-sided proptosis, a relative afferent pupillary defect (RAPD) and reduced color vision OD. Automated perimetry demonstrated constriction of the right visual field. Magnetic resonance imaging (MRI) revealed an enhancing mass along the greater wing of the right sphenoid bone extending through the superior orbital fissure into the posterolateral orbit with an accompanying dural tail that extended towards the frontal bone in the right middle cranial fossa. Due to compressive optic neuropathy, multidisciplinary tumor resection and reconstruction was recommended.

The tumor was resected via a right FTOZ craniotomy. The orbital roof and lateral wall were removed to the extent of the superior and inferior orbital fissures. The optic canal was exposed with the help of the operating microscope and diamond burr drill. The dural component of the tumor was also resected. After dural reconstruction, the orbital roof and lateral walls were reformed with a manually fashioned porous polyethylene implant (Medpor Titan Cranial Curve, Stryker Craniomaxillofacial, Kalamazoo, MI). The implant was fixated anteriorly at the remaining superolateral orbital rim, abutting the remaining frontal bone along the medial portion of the orbital roof and zygomatic bone along the inferior portion of the lateral wall.

**Figure 1:**
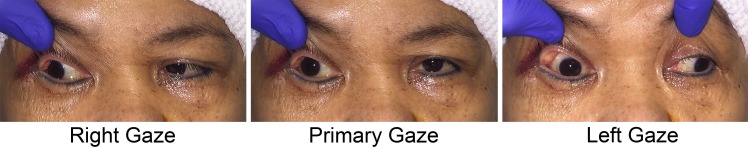
Case 1 with restrictive strabismus after frontotemporal-orbitozygomatic craniotomy for a right sphenoid wing meningioma On postoperative day one, an exotropia of the right eye in primary gaze is noted, and an inability to adduct the eye beyond the midline is demonstrated in Case 1.

**Figure 2:**
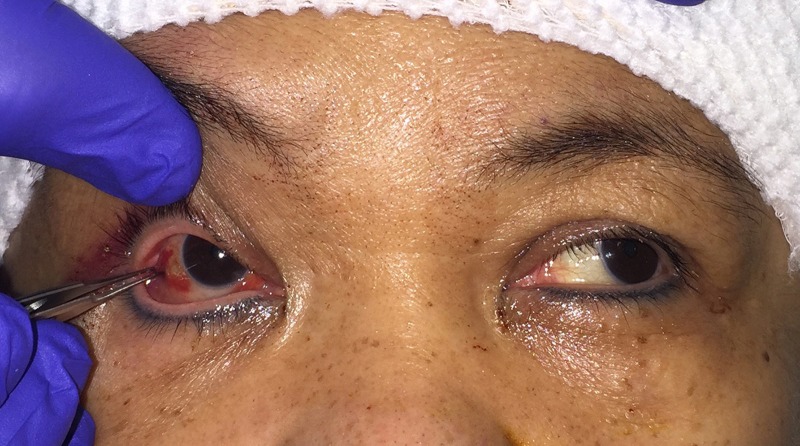
Forced duction testing Forced duction testing (FDT) is performed by applying topical anesthetic drops to the globe, grasping the muscle at its insertion or the corneo-scleral limbus with toothed forceps and rotating the globe in the direction of restriction. In Case 1, positive FDT is demonstrated by the inability to passively adduct the right eye.

**Figure 3:**
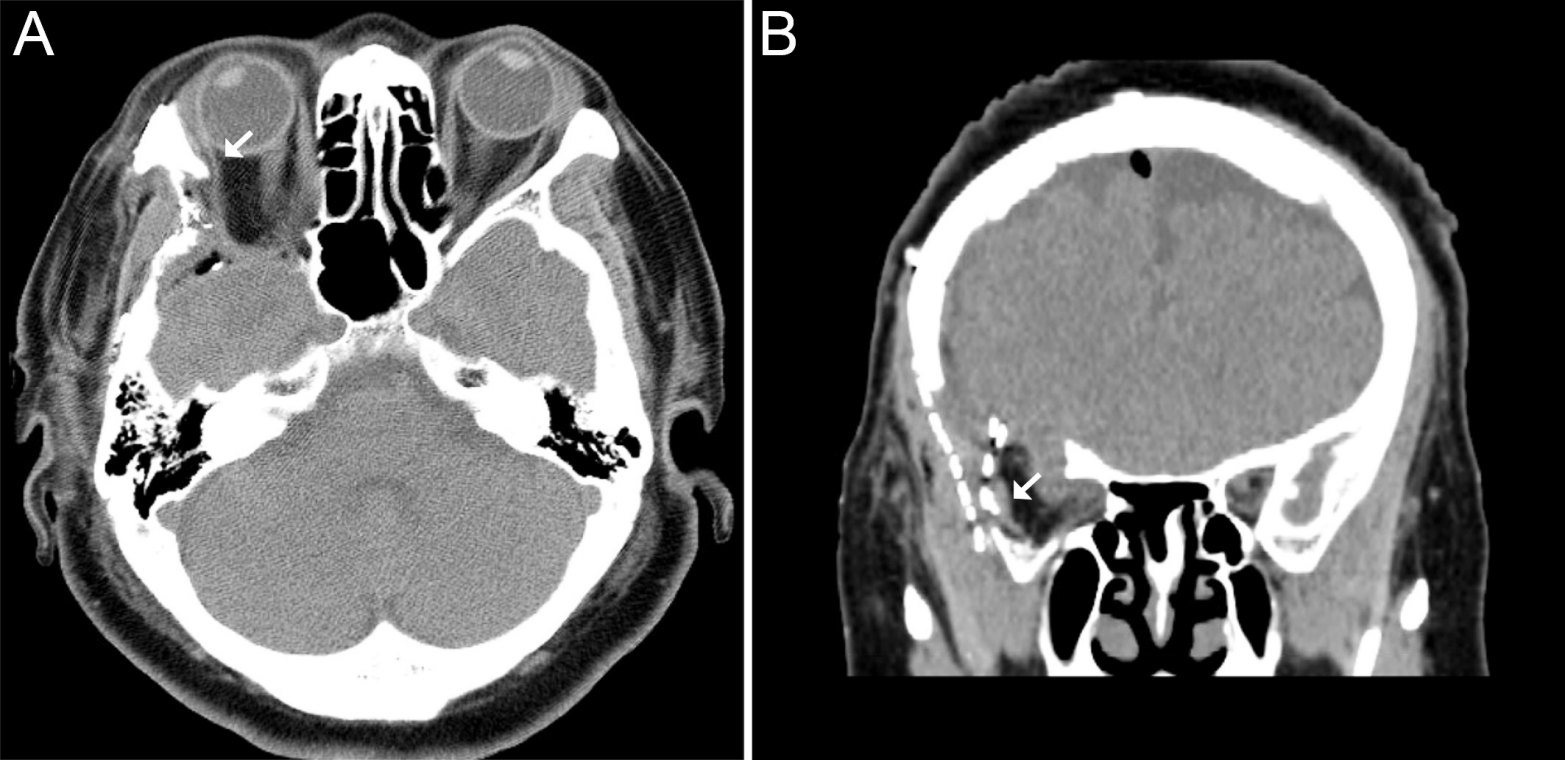
Computed tomography of the orbits in Case 1 Computed tomography of the orbits in Case 1 demonstrates impingement of the right lateral rectus muscle by a residual lip of zygomatic bone on axial image (A, arrow), as well as the alloplastic implant on coronal image (B, arrow).

On postoperative day one, the patient complained of binocular horizontal diplopia, and an examination demonstrated the inability to adduct the right eye beyond the midline (Figure 1). Bedside forced duction testing was performed by applying topical anesthetic drops to the right globe, grasping the muscle at its insertion or the corneo-scleral limbus with toothed forceps and rotating the globe in the horizontal plane (Figure 2). The eye could not be passively adducted, confirming restriction of the lateral rectus muscle. Postoperative computed tomography (CT) imaging suggested impingement of the right lateral rectus muscle by a sharp ridge of zygomatic bone at the prior level of the inferior orbital fissure (Figures 3A-3B). This was compounded by the inward curvature of the posterolateral extent of the implant, likely contributing to further alteration of the muscle vector. Forty-eight hours later, the patient was returned to the operating room for an orbitotomy through an upper eyelid crease incision. The sharp edge of zygomatic bone was removed. The posterolateral aspect of the implant was directed more laterally and a silicon sheet was placed between the lateral aspect of orbital soft tissue and the reconstructed lateral wall. Intraoperative forced duction testing confirmed release of the lateral rectus muscle.

**Figure 4:**
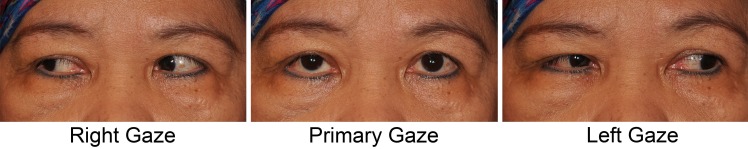
Case 1 after surgical revision for restrictive strabismus via orbitotomy In Case 1, the impingement of the lateral rectus muscle by bone and the implant were surgically addressed and at one month the patient is orthotropic in primary gaze with normal extraocular motility.

Twenty-four hours after reoperation, globe restriction had grossly resolved. Four weeks later, BCVA was 20/20 OD, both eyes were orthophoric, extraocular movements were full, globe projection was symmetric, and there was complete resolution of optic neuropathy (Figure 4).

Case 2

A 36-year-old woman with a WHO grade I SWM presented due to three months of progressive left-sided proptosis. An examination revealed BCVA of 20/20 in the left eye (OS), normal pupillary responses, full extraocular motility, and 3 mm of left-sided proptosis with supero-temporal periorbital fullness. Magnetic resonance imaging demonstrated an enhancing extra-axial mass beneath the inner table of the left frontal bone extending inferiorly to the sphenoid bone through the superior orbital fissure with involvement of the supero-lateral portion of the left extraconal orbit. A left FTOZ craniotomy was performed. The tumor was resected and the orbital roof and lateral wall were reconstructed using a porous polyethylene implant (Medpor Titan Cranial Curve, Stryker Craniomaxillofacial, Kalamazoo, MI) as described in the previous case.

**Figure 5:**
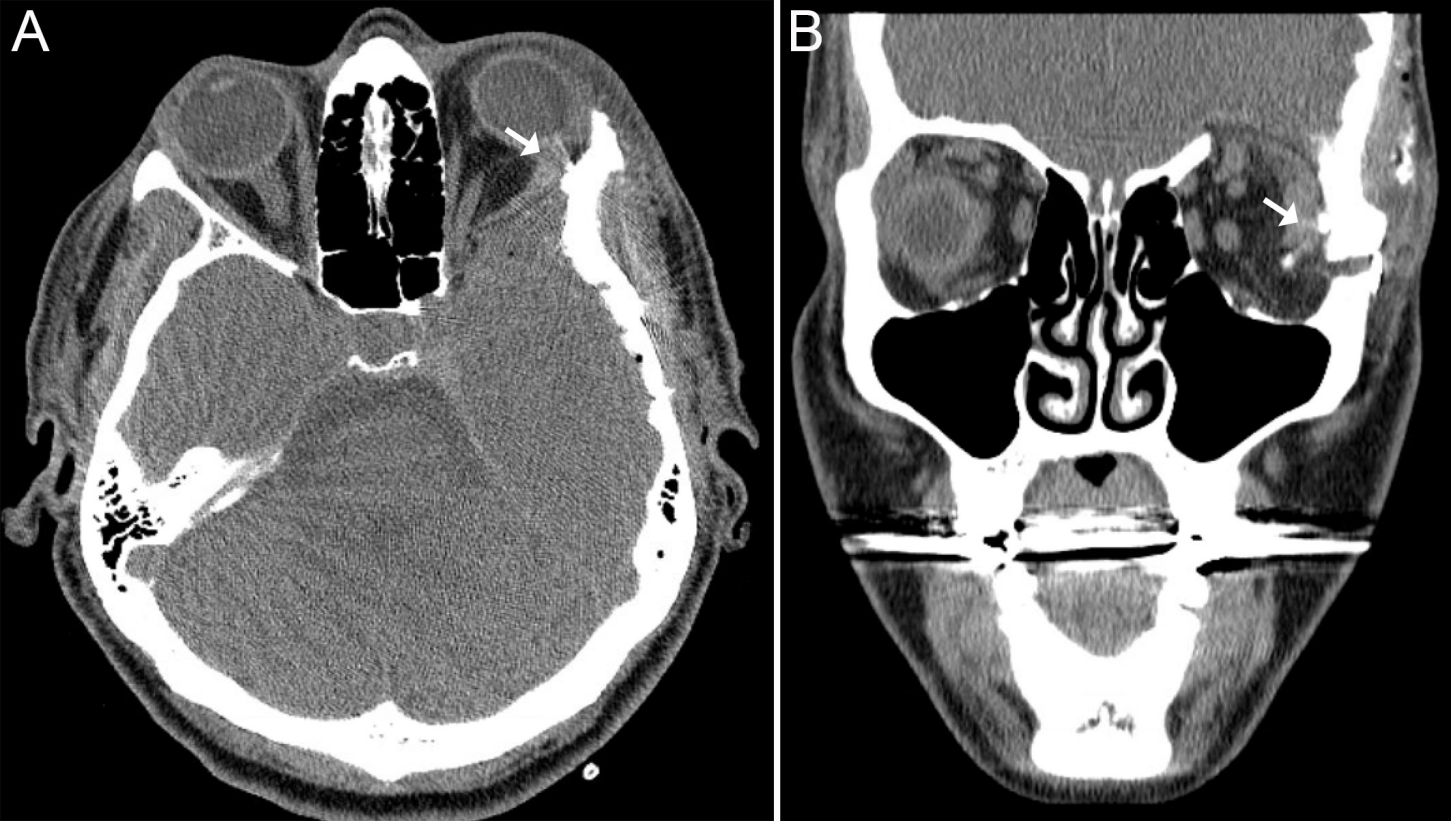
Computed tomography of the orbits in Case 2 Computed tomography of the orbits in Case 2 demonstrates impingement of the left lateral rectus muscle between the residual zygomatic bone and alloplastic implant on axial image (A, arrow) and coronal image (B, arrow). The axial image highlights the abnormal course of the muscle.

On postoperative day one the patient was exotropic and with restriction adduction on the left side. Forced duction testing was positive for a restricted left lateral rectus muscle. A postoperative CT scan showed impingement of the lateral rectus muscle between the implant and remaining zygomatic bone (Figures 5A-5B). She was returned to the operating room where, via an upper eyelid crease incision, the impinged lateral rectus was released and a silicone sheet was placed between the rectus muscle and lateral wall. Intraoperative forced duction testing confirmed release of the lateral rectus muscle.

Twenty-four hours after reoperation, the extraocular movements were grossly normalized. At six weeks, the examination demonstrated a BCVA of 20/20 OS, orthophoria with full extraocular movements, and symmetric globe projection.

## Discussion

Although neurosurgical access to SWMs has improved with the FTOZ approach, ophthalmologic risks associated with resection and reconstruction of the roof and lateral wall of the orbit include enophthalmos, vision loss, ocular misalignment, and diplopia. Earlier studies attributed extraocular muscle restriction to physical injury of the muscle or respective cranial nerve during surgery, postoperative edema, and soft-tissue adhesion to the lateral wall osteotomy [3-5]. Liu, et al. reported limitation of extraocular muscle in 40.5% (15/37) of patients after a pterional approach, which was transient in nature in 73.3% of cases and attributed to excessive traction during surgery [4]. In a review of 75 patients who underwent pterional surgery, Youssef, et al. identified two (2.6%) cases of extraocular movement limitation; however, the mechanism was not reported [6].

We report two cases of restrictive strabismus resulting in binocular horizontal diplopia immediately after FTOZ craniotomy for resection of SWM where there was extensive removal of the roof and lateral wall of the orbit due to bone infiltration and hyperosotosis by tumor. Both patients had limited adduction with a corresponding exotropia in primary gaze and a positive forced duction test (FDT) postoperatively. Vertical diplopia following craniotomy via a pterional approach has also been observed [5-7]. In a series by Desai, et al., the proposed mechanism was adhesion of the extraocular muscle to the orbital roof, reducing its ability to rotate the globe [5]. Similarly, Kaeser and Klainguti attributed vertical diplopia to the adhesion of the superior rectus/levator palpebrae complex to the orbital roof [7].

The lateral rectus muscle arises from the annulus of Zinn, spanning the superior orbital fissure. It then travels anteriorly, parallel to the greater wing of the sphenoid. Including its tendinous attachment to the globe, the lateral rectus has the second longest course of an extraocular muscle after the superior oblique. Stripping of the periorbita for tumor resection will expose the muscle and surrounding orbital fat potentially disrupting the orbital septa and glide plane of the muscle. Moreover, the muscle can be prone to mechanical restriction from alterations in the bony anatomy and/or implant placed for reconstruction that result in a step-off or ridge as demonstrated in this series. Desai, et al. also did report adhesion of the lateral rectus muscle to the osteotomy following lateral wall removal, further supporting the significance of the surgical anatomy in this complication [5].

Certainly, the prevention of postoperative restrictive strabismus is preferred. This begins with an intraoperative inspection of the resection site to ensure a bony step-off has not been inadvertently created that may impinge the course of the lateral rectus muscle. Similarly, fashioning of the implant used for orbital wall reconstruction must be performed with care to not result in a ridge that may impede the action of the muscle. Finally, the patient must be appropriately draped at the beginning of surgery to allow intraoperative visualization of both eyes for comparing globe position and performing the FDT to check for restrictive strabismus.

The FDT has been used for many years to differentiate between paretic and restrictive cause of reduced extraocular movement [8]. A positive FDT indicates the presence of mechanical factors as a cause of limited ocular motility. Oculoplastic surgeons routinely perform the FDT at the start of an orbital fracture repair when preoperative limitation is noted and indicative of muscle entrapment. Such testing allows one to know when the entrapped muscle has been adequately released with improvement of the FDT during the course of surgery.

Should restrictive strabismus be noted after the completion of FTOZ craniotomy, the timing of surgical intervention to address diplopia remains inconclusive. Some authors have reported conservative management, as well as delayed surgical intervention with varied outcome [4-5]. The ophthalmic literature supports early rather than late correction. Lisman, et al. have reported that diplopia from the entrapped muscle may become persistent without immediate surgical intervention [9]. This is attributed to ischemic muscle necrosis that leads to fibrosis and adhesion of the muscle to the periosteum with resultant permanent diplopia. Jordan, et al. reported that patients who underwent surgical exploration and repair within four days of extraocular muscle entrapment had complete resolution of diplopia and no permanent deficit [10]. Thus, should restrictive strabismus be noted in the immediate postoperative period after FTOZ craniotomy, we would recommend prompt surgical revision. Otherwise, there is potential risk of muscle compromise akin to the white-eyed, orbital floor blowout fracture seen in children.

## Conclusions

In summary, extraocular muscle restriction can occur after FTOZ craniotomy and orbital wall reconstruction for SWM with varied causes. In this report of two cases, it was attributed to impingement of the lateral rectus muscle by the postoperative bony anatomy and/or alloplastic implant resulting in restrictive strabismus and binocular diplopia. Employing intraoperative forced duction testing can allow for immediate recognition of the restriction and its correction prior to the closure of the craniotomy wound and is now part of our routine practice. Should restrictive strabismus be noted intraoperatively or in the immediate postoperative period, prompt surgical revision would be recommended.
